# Operative versus non-operative management following Rockwood grade III acromioclavicular separation: a meta-analysis of the current evidence base

**DOI:** 10.1007/s10195-011-0127-1

**Published:** 2011-02-23

**Authors:** Toby O. Smith, Rachel Chester, Eyiyemi O. Pearse, Caroline B. Hing

**Affiliations:** 1 Faculty of Medicine and Health Science, University of East Anglia, Norwich, UK; 2 St George’s Hospital, London, UK

**Keywords:** Acromioclavicular, Dislocation, ACJT, Rockwood type, Systematic review

## Abstract

**Background:**

Whilst there is little debate over the treatment of Rockwood grade V and VI acromioclavicular dislocation, the management of grade III acromioclavicular dislocation remains less clear. The purpose of this study was to compare the clinical outcomes of patients managed operatively and non-operatively following grade III acromioclavicular dislocation.

**Materials and methods:**

A systematic review of published and unpublished material was conducted. All included studies were reviewed against the PEDro appraisal tool. Where appropriate, a meta-analysis of pooled results was conducted.

**Results:**

Among 724 citations, six studies met the eligibility criteria. All six studies were retrospective case series (level 4 evidence). The findings of this study indicated that operative management of grade III acromioclavicular dislocation results in a better cosmetic outcome (*P* < 0.0001) but greater duration of sick leave compared to non-operative management (*P* < 0.001). There was no difference in strength, pain, throwing ability and incidence of acromioclavicular joint osteoarthritis compared to non-operative management. Only one study recorded and showed a higher Constant score for operative management compared to non-operative management (*P* = 0.003).

**Conclusions:**

There is a lack of well-designed studies in the literature to justify the optimum mode of treatment of grade III acromioclavicular dislocations.

## Introduction

Rockwood’s classification of acromioclavicular dislocation is based on the degree and direction of clavicular displacement [1]. Grades I and II are benign and are widely regarded as best managed conservatively [2, 3]. There is a general consensus that type V and VI lesions should be treated operatively [2, 4]. However, there remains controversy over the optimal management strategy for grade III and IV injuries [4–7]. Grade III is classified as a superior displacement of the lateral end of the clavicle of one clavicular diameter or 1 cm on the anteroposterior radiograph, whilst grade IV is described as a separation of the acromioclavicular joint with the distal clavicle displaced posterior into the trapezial fascia [6, 7]. In both grades the acromioclavicular and coracoclavicular ligaments are torn.

Advocates of non-operative treatment suggest that patients often regain excellent clinical results and painless shoulder function, although for some there is the potential for chronic instability and pain [8, 9]. Alternatively, operative treatment strategies are able to address these shortcomings, but occasionally compromise shoulder function [2, 8].

Given this degree of equipoise, the purpose of this study was to compare the clinical outcomes of patients managed operatively and non-operatively following grade III acromioclavicular dislocation.

## Materials and methods

### Study eligibility

To be eligible for inclusion in the systematic review, studies had to compare operative to non-operative management following an acute, closed grade III acromioclavicular dislocation. Studies had to report at least one outcome of interest (see below). All randomised controlled trials (RCTs) and non-randomised controlled trials (nRCT) were included.

All included studies reported that all patients recruited gave informed consent prior to being included. All studies were authorized by a local ethical committee, and performed in accordance with the ethical standards of the 1964 Declaration of Helsinki, as revised in 2000.

### Search strategy

The electronic databases: MEDLINE, Embase, Cinahl, Ahmed, Cochrane library and Scopus were searched from their inception to 1st May 2010 in accordance to PRISMA guidelines [10]. A secondary search was conducted reviewing unpublished literature databases including: Greynet, SIGLE, National Technological Information Service, British Library Integrated catalogue, Current Controlled Trials and the Cochrane Central Register of Controlled Trials.

In order not to omit any important papers, a broad search was initially undertaken using the MeSH terms and Boolean operators (“acromi$” OR “acromioclavicular”) AND (“injur$” OR “disrupt$” OR “dislocat” OR “subluxat$” OR “ruptur$”) AND (“operat$” OR “surg”) AND (“conservat$” OR “non-surg$” OR “immobilis$” OR “rehabilit$” OR “physical therapy” OR “physiotherapy”).

The reference lists of all potentially eligible studies were reviewed. Finally, the corresponding authors of all eligible studies were contacted and asked to review the search results to identify any studies which may have been initially missed.

### Study identification

Two reviewers (TS, CH) independently screened the titles and/or abstracts of all identified citations against the inclusion and exclusion criteria. The full texts of all potentially eligible studies were obtained. These were then reviewed against the eligibility criteria before inclusion in the review.

### Data extraction

One reviewer extracted all the data onto a pre-defined database (CH). This was then independently verified by a second reviewer for accuracy (RC). Data collected included patients’ characteristics, study design, interventions, follow-up periods and relevant outcomes.

### Methodological appraisal

Study methodological assessment was evaluated using the PEDro score. This is an eleven-item critical appraisal tool which assesses documentation of eligibility, subject allocation and randomisation, subject assessment and blinding, subject follow-up, data assessment and analysis. This has previously been demonstrated to be a reliable and valid scoring system [11, 12]. The critical appraisal was conducted by one reviewer (CH), and independently verified by a second reviewer (RC).

### Outcomes of interest

The primary outcome was the Constant score [13]. Secondary outcomes included: duration of sick leave, strength, pain, cosmetic outcome, implant failure, infection rate, throwing ability, loss of reduction of anatomical position, ossification of the coracoclavicular ligament, range of motion, and the incidence of acromioclavicular joint osteoarthritis (OA).

### Data analysis

An assessment of study heterogeneity was made by observing for population or interventional differences between the studies from the data extraction tables. Secondly, statistical heterogeneity was evaluated using the chi^2^ (*χ*^2^) test and *I*^2^ statistics. For outcomes when *I*^2^ and*χ*^2^ were less than 20% or *P* < 0.05, a fixed-effects model was adopted. When these assumptions were not met, a random-effects model was adopted. A meta-analysis was conducted where appropriate to pool outcomes. For dichotomous outcomes, the effects measure was the risk difference (RD). For continuous outcome measures, the effect measure was mean difference (MD) or standardised mean difference (Std MD). In each case, a *P* < 0.05 was considered statistically significant, and 95% confidence intervals (CI) were calculated.

The principal analysis was to compare outcomes between operative and non-operative management of acromioclavicular joint grade III dislocations. A secondary analysis included a sensitivity analysis to compare outcomes for RCTs only. Publication and small study bias was assessed using a funnel plot. All meta-analyses were performed using the Review Manager software (RevMan Version 5.0; Nordic Cochrane Centre, Copenhagen, Denmark) and the Mantel–Haenszel method [14].

## Results

### Search strategy results

**Fig. 1:**
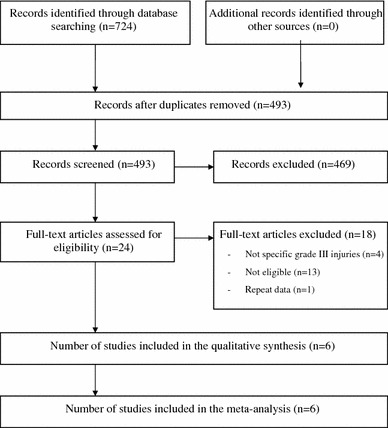
PRISMA chart illustrating the results of the search strategy

**Fig. 2:**
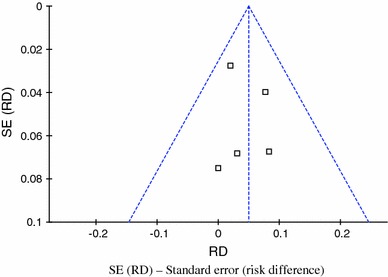
Funnel plot illustrating publication bias using the cosmetic results outcome measure

A total of 724 citations were identified (Fig. 1). Twenty-four were identified as potentially relevant. On second review, thirteen were deemed not appropriate, whilst one study reported the outcomes of the same cohort in two publications [15, 16]. The most recent version of this paper was included in the review [15]. Four studies did not clearly define the grade of acromioclavicular displacement [17–20]. To minimise review heterogeneity, these studies were excluded, leaving six eligible studies. All were retrospective case series. The funnel plot of infection rate indicated mild evidence of small study exclusion and publication bias (Fig. 2).

### Methodological quality

The findings of the PEDro critical appraisal indicated that the methodological quality of the current evidence base was poor (Table 1). Although all studies clearly defined their study participants, only two studies demonstrated baseline comparability between the operative and non-operative groups [21]. Furthermore, no study randomised their patients to the allocated intervention. No study based their sample size on a power calculation. Whilst it may have been impractical to blind subjects or clinicians to treatment allocation, no study blinded their assessors during the investigations. Although subject drop-out was more than 85% in all but two studies, no study analysed their results by intention-to-treat principles, or adjusted their results to estimate this missing data. Nonetheless, all clearly described their results and appropriately used descriptive and inferential statistical tests to analyse their cohorts.

**Table 1:** PEDro score

	Calvo et al. [8]	Fremerey et al. [15]	Galpin et al. [26]	Gstettner et al. [22]	Press et al. [21]	Taft et al. [9]
Eligibility criteria	1	1	1	1	1	1
Random allocation	0	0	0	0	0	0
Concealed allocation	0	0	0	0	0	0
Baseline comparability	1	1	1	1	0	0
Blind subject	0	0	0	0	0	0
Blind clinician	0	0	0	0	0	0
Blind assessor	0	0	0	1	0	0
Adequate follow-up (≥85%)	1	0	0	0	0	1
Intention-to treat analysis	0	0	0	0	0	0
Between-group analysis	1	1	1	1	1	1
Point estimates and variability	1	1	1	1	1	1
Total score	5	4	4	5	3	4

1: criterion satisfied; 0: criterion not satisfied

### Study characteristics

In total, 380 patients were included in the review (Table 2). The operative management cohort consisted of 195 shoulders, 125 males and 15 females with a mean age of 24.4 [standard deviation (SD) = 4.5] years. The non-operative group consisted of 185 shoulders, 96 males and 13 females with a mean age of 27.8 (SD = 6.1) years. One study did not document the cohort age or gender, and therefore total numbers for age and gender are incomplete [9]. Five studies solely evaluated outcomes in patients with Grade III Rockwood injuries. One study’s cohort consisted of 78% grade III injuries, and 22% grade V injuries [15]. Given this high proportion, and since this study provided some outcomes based on grade of injury separately, this study was included in the review.

**Table 2:** Study characteristics

Study	Study	Sample		Age (years)		Gender (m/f)		Gd of ACJ dis.	Surgical mgmt	Non-surgical mgmt	Mean follow up (years)
		Op	NOp	Op	NOp	Op	NOp				
Calvo et al. [8]	Retro	32	11	40	35	27/5	11/0	Type III	Phemister technique	Sling—type not specified. Physiotherapy—type not specified	Op: 123 m NOp: 41 m
Fremerey et al. [15]	Retro	51	46	33.7	35.9	48/3	39/7	77 Gd III 20 Gd V	PDS	Physiotherapy exercises	Op: 6.1 NOp: 6.5
Galpin et al. [26]	Retro	16	21	29	37	16/0	17/3	Gd III	Bosworth	Sling—type not specified. Strength and ROM exercises	Op: 35.0 m NOp: 33.7 m
Gstettner et al. [22]	Retro	28	22	37.2	36.2	25/3	20/2	Gd III	Hook plate	Sling—type not specified. Physiotherapy—type not specified	34 m
Press et al. [21]	Retro	16	10	30.7	49.6	12/4	9/1	Gd III	Weaver–Dunn procedure	Sling—type not specified. Rehabilitation—not specified	32.3 m
Taft et al. [9]	Retro	52	75	N/S	N/S	N/S	N/S	Gd III	Bosworth screw or 1–3 Steinmann pin fixation	Sling, Kenny–Howard splint, taping or cast. Mobilisation exercises	Op: 10.8 Nop: 9.5

*av* average, *Co* conservative management, *Comp* complete, *Dis* disruption, *exs* exercises, *Gd* grade, *gp* group, *immob* immobilised, *inj* injury, *K wire* Kirschner wire, *Lig* ligament, *m* months, *Mgmt* management, *ND* Not documented, *Op* operative management, *physio* physiotherapy, *Pros* Prospective, *rec* recreational, *recon* reconstructed, *Ret* retrospective, *RTA* road traffic accident, *Sed* sedentary, *sh* shoulder, *wks* weeks

The operative procedures were clearly described in all papers. Five studies included fixation using Kirschner wire or screw fixation methods, whilst one study used hook plates as the form of fixation [22]. All studies reported repair of coracoclavicular and acromioclavicular ligaments using sutures. The non-operative management adopted was poorly described. All subjects were immobilised using a sling of some description. However, Taft et al. [9] reported immobilising patients using a taping technique or cast, but did not specify the method of application. Immobilisation varied between the studies from 2 weeks [8] to 4 weeks [9]. The remaining papers reported immobilising patients until pain and symptoms had resolved. Following this, subjects commenced range of motion and/or strength rehabilitation programmes, but this was not described in detail in the studies. The follow-up period ranged from 32 months [21] to 10.8 years [9].

### Meta-analysis

**Fig. 3:**
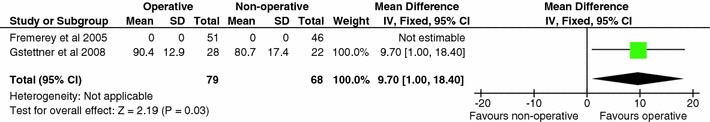
Forest plot illustrating constant score

**Fig. 4:**
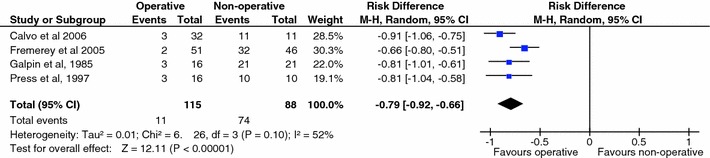
Forest plot illustrating cosmetic outcome

**Fig. 5:**
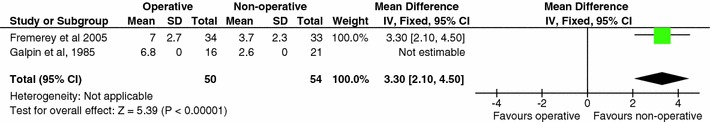
Forest plot illustrating duration of sick leave

**Fig. 6:**
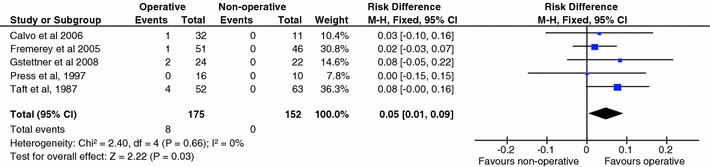
Forest plot illustrating infection rate

The results of the meta-analysis are shown in Table 3. The primary outcome of this study was the Constant score. This revealed that there was a significantly better functional outcome following operative compared to non-operative management of grade III acromioclavicular separation (MD = 9.70; 95% CI: 1.00, 18.40; *P* = 0.03; Fig. 3). However, this is based on the complete data from one study [22]. There was no statistically significant difference between the interventions in respect to strength, pain, throwing ability, loss of anatomical reduction, ossification of the coracoclavicular ligament or acromioclavicular joint osteoarthritis (*P* > 0.05). There were significantly poorer cosmetic results following non-operative management (RD = 0.64; 95% CI: 1.09, 0.19; *P* < 0.0001; Fig. 4). The results also suggested that there was a significantly greater duration of sick leave following operative management compared to non-operative management (MD = 3.3; 95% CI: 2.10, 4.50; *P* < 0.001; Fig. 5). Although a relatively low incidence, unsurprisingly, the infection rate was significantly higher in the operative compared to the non-operative group (RD = 0.05; 95% CI: 0.01, 0.09; Fig. 6).

**Table 3:** Results of the meta-analysis

Outcome	Studies	Effect estimate	*P*-value	Heterogeneity	
				*I* ^2^	Chi^2^ (*P* value)
Duration of sick leave	2	3.30 (2.10, 4.50)	<0.0001	NE	NE
Constant score	2	9.70 (1.00, −18.50)	0.03	NE	NE
Throwing ability	3	−0.00 (−0.15, 0.15)	0.98	0	0.57
Strength (≥90% normal)	2	−0.01 (−0.12, 0.11)	0.90	0	0.82
Strength (≤70% normal)	2	0.35 (0.04, 3.51)	0.37	0	0.88
No pain	2	0.90 (0.33, 2.41)	0.83	0	0.60
Severe pain	2	−0.00 (−0.06, 0.06)	0.95	0	0.97
Poor cosmetic outcome	4	−0.79 (−0.92, −0.66)	<0.0001	52	0.10
Tenderness over the acromioclavicular joint	2	0.08 (−0.23, 0.40)	0.61	75	0.05
Implant failure	2	0.04 (−0.05, 0.13)	0.42	0	0.91
Infection	5	0.05 (0.01, 0.09)	0.03	0	0.66
Loss of anatomical reduction	2	0.50 (−1.07, 0.52)	0.50	98	<0.0001
Ossification of the coracoclavicular ligament	2	0.17 (−0.32, 0.66)	0.50	82	0.02
Acromioclavicular joint osteoarthritis	3	0.12 (−0.21, 0.46)	0.46	89	0.001

*NE* not estimable

One study assessed the effect of range of motion. Fremerey et al. [15] reported no substantial difference between the interventions, with two patients demonstrating a loss of abduction and external rotation following operative management compared to one patient following non-operative rehabilitation (*P* > 0.05). Finally, Press et al. [21] reported loss of reduction from the anatomical position. They documented that two patients following operative management presented with loss of reduction, compared to no cases following non-operative management.

### Sensitivity analysis

It was not possible to undertake a sensitivity analysis since none of the studies included in the meta-analysis were randomised controlled trials.

## Discussion

The principal finding of this study was that, for the majority of outcomes, there was no statistically significant difference in clinical or radiological outcomes between operative and non-operative management for this patient group. Nonetheless, there was some evidence to suggest that operative management provided a significantly better Constant score compared to non-operative following grade III acromioclavicular dislocation, but this was based on the results from a single study. Non-operative management was associated with significantly poorer cosmetic outcome but less sick leave compared to operative management (*P* < 0.001).

The current evidence base presented with a number of methodological limitations, including not randomising patients to group allocation, permitting allocation bias [23], and not blinding assessors to subject groups, therefore increasing the risk of assessment bias [24]. Finally, the studies did not base their sample sizes on power calculations, increasing the risk of a type II statistical error due to an insufficient sample size [25]. Accordingly, future robust, well-designed RCTs are required to improve the currently poor evidence base in order to determine the optimal management strategy for patients following grade III acromioclavicular separation.

A previous meta-analysis by Philips et al. [6] ultimately advised against surgical treatment following grade III acromioclavicular separation. This review differed to the previous review as it specifically included only those studies with cohorts of predominantly grade III acromicoclavicular separation. Furthermore, with the advantage of time, we have been able to include a number of studies which have recently been published on this topic. Whilst there is agreement with some of Philips et al.’s [6] conclusions, this study concludes, with some reservations, that there is little difference in the outcome of operative and non-operative management for patients following grade III acromioclavicular separation, with the exception that non-operative management provides cosmetically poorer outcomes. A more recent paper [22] has shown that maintenance of reduction is possible with the operative group having a statistically better outcome than the non-operative group. As operative techniques improve, there may be a paradigm shift from the historically poor results of fixation with K-wires.

The mechanism of injury appeared similar among the studies, with a combination of sporting, accidental and occupation trauma as the associated factor. Few studies distinguished whether upper limb dominance was a factor in outcome. This may have a been particularly important confounding variable for functional-based outcomes and return to sports measures, where those with a dominant limb injury may present with poorer outcomes—particularly during early review—compared to non-dominant limb injury. A further confounding factor which may have affected outcome was time from injury to surgery. Rolf et al. [2] reported that those patients who had an acute acromioclavicular reconstruction after trauma reported significantly better functional outcomes and patient satisfaction rates as well as lower complication rates compared to patients with delayed reconstruction. Whilst the four studies reported that all operations were acute, the duration from injury to surgical reconstruction was not clearly stated in the papers of Galphin et al. [26] or Taft et al. [9]. Finally, to the study’s credit, the follow-up period of the evidence base was reasonable, providing some evidence for detecting late failures and longer-term outcomes.

The results of this meta-analysis indicate that there was no significant difference in respect to maintenance of anatomical reduction between operative and non-operative management of grade III acromioclavicular separation (*P* = 0.15). Calvo et al. [8] acknowledged that complete reduction may not necessarily be a pre-requisite for optimal functional outcome [6, 8, 27]. They suggested that the rationale of surgical reconstruction to achieve anatomic alignment for full functional recovery may not always be achieved following grade III acromioclavicular separation [8]. Thus, anatomical reduction alone cannot justify operative intervention. However, the method of assessing anatomical alignment was unclear from the included studies. Previous authors have argued that only by assessing the acromioclavicular joint with stress radiography can anatomical position be determined [28]. Accordingly, future study is recommended to determine the optimal method of radiographic evaluation of acromioclavicular displacement following operative and non-operative management strategies.

The current meta-analysis suggests that there was no difference in the incidence of OA or ossification of the coraclavicular ligament between the two management strategies. Authors such as Calvo et al. [8] have suggested that the incidence of OA changes may be related to the surgical manipulation and inability to maintain reduction, whilst ossification of the coracoclavicular ligaments has been associated with the manipulation of ligament tissue when attempting to repair it [8, 29]. Fremerey et al. [15] and Taft et al. [9] suggested that post-traumatic OA in surgically managed patients is related to the unphysiological contact of traumatised joint surface and subsequent joint cartilage injury.

Several authors have suggested that surgical reconstruction should be advocated for those patients who have physically demanding occupations or sporting interests. However, since the mean age of each study’s cohort was under 28 years, and the mechanism of injury was largely sporting or occupationally related, there was little evidence to substantiate this claim based on clinical outcomes. Furthermore, since this study suggested that duration of sick leave was significantly higher following non-operative procedures, and that there was no significant difference in strength outcomes, then non-operative management may be seen as superior to manage this patient group. For those patients who carry heavy weights on their shoulders, such as soldiers carrying rucksacks, operative intervention may be indicated to prevent anatomical deformities from affecting return to normal activities.

The literature poorly described the non-operative management strategies used. Historically, various straps, harnesses, casting techniques and traction methods have been used as part of closed reduction [30–34]. Currently, there appears greater support for the use of internal rotation slings. Since non-operative management strategies were not clearly defined, it remains unclear as to whether there was a variation in these strategies between the studies. Furthermore, it also remains unclear as to whether clinical outcomes are affected by the type of rehabilitation programme adopted, immobilisation method or period of immobilisation.

As Gstettner et al. [22] acknowledged, the disadvantage of all operative strategies is the risk of complications. This was mirrored by our study, which demonstrated a significantly higher risk of infection following surgical management compared to non-operative treatment (*P* = 0.03). However, the incidence of infection was relatively low following acromioclavicular surgery. There has been a paradigm shift in clinical practice. Earlier studies adopted Phemister fixation methods. This developed into a consensus of using Bosworth screw and then later Hook plate fixation methods [35–38]. Currently, TightRope fixation methods and biodegradable slings have been introduced [39, 40]. Whilst clinical differences between operative and non-operative strategies have evaluated previous surgical interventions, the comparison to biodegradable sling fixation is yet to be evaluated using a large, well-designed RCT.

Finally, no study compared cost-effectiveness with a formal economical evaluation. Since the meta-analysis indicated that whilst patients reported a shorter duration of sick leave following non-operative management, and that higher costs of hospitalisation, the operative procedure and prolonged rehabilitation are associated with this strategy, there initially appears to be greater support from an economic perspective for adopting a non-operative management strategy for this patient group. Formal health economical assessment is therefore imperative to assess the differences in this and clinical outcomes when developing the evidence base with well-designed, sufficiently powerful RCTs.

To conclude, based on the current evidence base, operative management of grade III acromioclavicular dislocations results in a better cosmetic outcome (*P* < 0.0001) but a greater duration of sick leave (*P* < 0.001) compared to non-operative management. There was no difference between the two interventions in terms of strength, pain and throwing ability (*P* > 0.05).
